# A Polarization‐Modulated Information Metasurface for Encryption Wireless Communications

**DOI:** 10.1002/advs.202204333

**Published:** 2022-10-17

**Authors:** Hai Lin Wang, Hui Feng Ma, Tie Jun Cui

**Affiliations:** ^1^ State Key Laboratory of Millimeter Waves School of Information Science and Engineering Southeast University Nanjing 210096 China; ^2^ Institute of Electromagnetic Space Southeast University Nanjing 210096 China

**Keywords:** encryption, metasurfaces, polarization, programmable, wireless communications

## Abstract

Programmable and information metasurfaces have shown great potentials in wireless communications, but there are few reports on encrypted communications. In this paper, a programmable polarization‐modulated (PoM) information metasurface is proposed, which can not only customize arbitrarily linearly polarized reflected waves, but also modulate their amplitudes in real time. Based on this feature, a physical‐level wireless communication encryption scheme is presented and experimentally demonstrated by introducing a meta‐key, which can be encrypted and sent by the programmable PoM information metasurface. To be specific, the key is encoded and concealed into different linear polarization channels, and then modulated and transmitted by the information metasurface at the transmitting end. At the receiving end, the modulated signal can be received and decoded by using a pair of polarization discrimination antennas. A wireless transceiver system is established to verify the feasibility of the scheme. It is shown that, once the meta‐key is obtained, the corresponding encrypted target information that has been sent to the user in advance can be recovered.

## Graphical abstract

A programmable polarization‐modulated (PoM) information metasurface based on amplitude‐phase‐joint‐coding technique is reported, which can not only achieve arbitrarily linearly polarized reflected waves, but also modulate their amplitude in real time. Furthermore, a meta‐key scheme based on the proposed programmable PoM information metasurface for wireless communication encryption is presented and demonstrated experimentally.

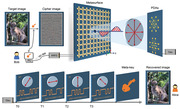

## 1 Introduction

Metasurfaces, which consist of two‐dimensional (2D) arrays of subwavelength planar metallic or dielectric meta‐atoms, have been extensively used for controlling the electromagnetic (EM) waves in multi‐degrees of freedom,^[^ ^1^, ^2^ ^]^ in which polarization modulation (PoM) is one of the important research topics.^[^ ^3^, ^4^, ^5^ ^]^ Many versatile PoM methods based on passive metasurfaces have been proposed by using the principle of phase control, including propagation phase,^[^ ^6^, ^7^, ^8^ ^]^ geometric phase,^[^ ^9^, ^10^, ^11^ ^]^ and their combination.^[^ ^12^, ^13^, ^14^, ^15^ ^]^ However, the functions of abovementioned passive metasurfaces cannot be changed once manufactured. Fortunately, by integrating with active components or phase‐change materials, tunable metasurfaces can be achieved to realize dynamical control of EM waves, which have been widely used in dynamically holographic imaging,^[^ ^16^, ^17^, ^18^ ^]^ reconfigurable intelligent surfaces,^[^ ^19^, ^20^, ^21^ ^]^ and so on.^[^ ^22^, ^23^ ^]^ In addition, these tunable metasurfaces were also used for dynamic manipulation of polarization state of EM waves or light, implementing versatile polarization controls,^[^ ^24^, ^25^, ^26^, ^27^, ^28^, ^29^, ^30^, ^31^ ^]^ but most of the existing works based on phase‐only control are restricted to the dynamic polarization conversion between co‐polarized waves, cross‐polarized waves, or linear‐circular polarized waves. Thus, it is still a challenge to achieve real‐time polarization manipulation with more degrees of freedom. In recent years, time‐varying metasurfaces have been proposed by applying a time‐varying periodic bias voltage to active components loaded on metasurfaces, which have shown extraordinary ability in controlling the amplitude and phase distributions of EM waves at different harmonic frequencies.^[^ ^32^, ^33^ ^]^ Since the amplitude and phase of EM waves can be simultaneously manipulated, time‐varying metasurfaces can achieve more powerful PoM functions, such as editing arbitrarily linear polarizations,^[^ ^34^ ^]^ or even the generation of arbitrary polarization,^[^ ^35^ ^]^ and linear‐nonlinear polarization syntheses.^[^ ^36^ ^]^ However, these time‐varying metasurfaces must be derived by continuously applying a time‐varying periodic bias voltage, and the additional spectrum resources will be inevitably occupied due to the generation of harmonics, which still has limitations in practical applications of PoM.

In addition, information security is another interesting topic and always plays an important role in people's daily life. Based on the technology of metasurfaces, some interesting works about holographic encryption have been reported,^[^ ^37^, ^38^, ^39^, ^40^ ^]^ which have shown great potential value in optical encryption systems. While, the far‐field wireless communication is another important application of human beings and has always been a hot research topic. In recent years, digitally programmable metasurfaces have been rapidly developed,^[^ ^41^ ^]^ which make a bridge between the physical world and information science, and have shown great advantages in digital wireless communication applications since the architecture of the conventional radio‐frequency (RF) transmitters can be greatly simplified by adopting this new technology.^[^ ^42^, ^43^, ^44^, ^45^, ^46^ ^]^ However, to the best of our knowledge, the abovementioned studies are limited to unprotected direct information transmission, and the research about information encryption technology based on this new‐type wireless communication is still very limited.

In this paper, we propose a programmable PoM information metasurface based on amplitude‐phase‐joint‐coding technique without involving time modulation, which can not only achieve arbitrarily linearly polarized reflected waves, but also modulate the amplitude of the reflected waves in real time. Furthermore, a wireless communication encryption scheme is proposed by introducing a meta‐key using the programmable PoM information metasurface. First, the target information is encrypted by using the meta‐key and sent to the user in advance, which can be implemented by previously reported space‐time‐coding digital metasurface.^[^ ^42^ ^]^ Then, the meta‐key is encoded and encrypted by concealing its codes into different polarization channels according to an encryption protocol, and sent to the user using the programmable PoM information metasurface at the transmitting end. At the receiving end, a pair of polarization discrimination antennas (PDAs) is designed to receive and decode the encrypted signals of meta‐key. Once the user gets the meta‐key, the encrypted target information can be recovered. As validation, a wireless transceiver system is established to experimentally demonstrate the modulation and demodulation processes of the meta‐key. The results show that the proposed programmable PoM information metasurface not only can achieve good polarization modulation performance, but also shows good potentials in the encryption communications, which can greatly improve the security of the wireless communications.

## 2 Wireless Communication Encryption Scheme Based on the Programmable PoM Information Metasurface

**Figure 1:**
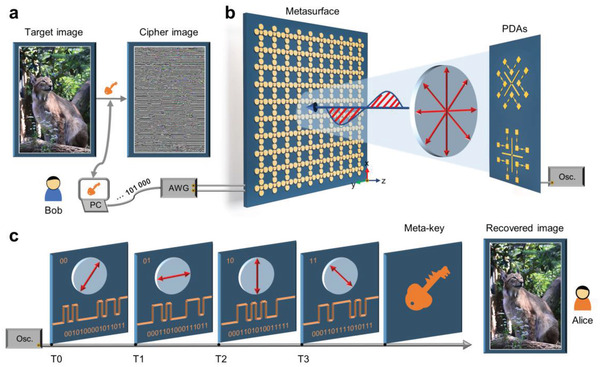
Conceptual illustration of the wireless communication encryption scheme based on the programmable PoM information metasurface. a) The target image is encrypted by using a meta‐key to generate a cipher image. b) A voltage‐controlled PoM metasurface encrypts and sends the meta‐key, and a pair of polarization discrimination antennas (PDAs) connected to an oscilloscope receives and identifies the signals. c) The meta‐key is decrypted by the signals received from four different polarization channels, and then the target cipher image can be recovered by the meta‐key. Photo credit: Hai Lin Wang, Southeast University.

**Figure** **1** shows a conceptual illustration of the wireless communication encryption scheme based on the programmable PoM information metasurface. At the transmitting end, a target image, such as a picture of the lynx, is encrypted by using a meta‐key and pre‐sent by Bob, as shown in Figure 1a. Therefore, even if the encrypted information is intercepted by a snooper, it is impossible for him to decipher it without the meta‐key. However, in order to enable Alice to get the correct target information, the meta‐key also needs to be sent to Alice securely except for encrypted target information, which is the focus of this work and can be implemented by using the programmable PoM information metasurface, as shown in Figure 1b. The metasurface is loaded with PIN diodes, and its reflection amplitude and phase in the vertical and horizontal direction can be independently controlled in real‐time by adjusting the state of PIN diodes. Therefore, when a 45°‐polarized incident wave illuminates to the metasurface, the polarization angle of the linearly polarized reflected wave can be arbitrarily edited. In addition, the amplitude of reflected wave in each polarization channel can also be further modulated. At the receiving end, two PDAs connected to an oscilloscope are used to receive signals (i.e., the modulated reflected wave), which can not only identify the polarization state of reflected wave, but also get the binary coding sequence based on amplitude shift keying in each polarization channel, as shown in Figure 1c. It is worth mentioning that these four coding sequences are sent by using the different polarization channels, and the binary codes of meta‐key are concealed in these sequences. Thus, Alice can decode the received signal to get the meta‐key according to the agreed encryption protocol, and then uses it to recover the target image. More details about the encryption and decryption processes can be found in Figure S1, Supporting Information.

## 3 Design of the Programmable PoM Information Metasurface

### 3.1 Simulation Results

**Figure 2:**
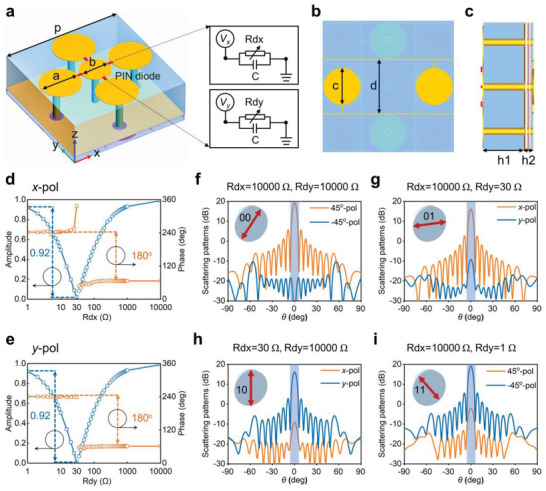
Prototype design and simulated results. a–c) The unit element of the PoM metasurface loaded with four PIN diodes: a) Front view, b) bottom view, and c) side view. d,e) The simulated reflection amplitudes and phases of the metasurface varying with *R*_d_ at 10 GHz: d) *x*‐polarized wave, e) *y*‐polarized wave. f–i) The simulated 2D far‐field radiation reflected patterns for four linear polarization states: f) 45° polarization, g) *x* polarization, h) *y* polarization, and i) −45° polarization.

**Figure** **2**a shows the unit element of the proposed programmable POM information metasurface, which is composed of three metal layers spaced by two dielectric substrates. Five metal circular patches loaded with PIN diodes on the top layer are connected to the feeder lines on the bottom layer via metalized through holes, so that the accurate bias voltage can be provided to the PIN diodes. The equivalent circuit of the PIN diode (Skyworks SMP1321‐040LF) is given in the right corner of Figure 2a. It is a parallel circuit of the fixed capacitor (*C* = 0.15 pF) and a variable resistor, in which the variable resistors along *x*‐ and *y*‐directions are defined as *R*_d_ *_x_* and *R*_d_ *_y_*, respectively. The values of *R*_d_ *_x_* and *R*_d_ *_y_* are controlled by row‐ and column‐controlled bias voltages *V_x_* and *V_y_*, respectively, and vary in the range of 1–10 000 Ω. The geometric parameters shown in Figure 2a are *p* = 14.2 mm, *a* = 4.4 mm, and *b* = 3.6 mm. Figure 2b shows the layout of feeder lines on the bottom layer, in which the diameter of the circular patch and distance between two feeder lines are *c* = 4 mm and *d* = 5.8 mm, respectively. The dielectric substrates are F4B (polytetrafluoroethylene) with relative permittivity of 2.2 and loss tangent of 0.001, whose thicknesses are *h*_1_ = 4 mm and *h*_2_ = 0.254 mm, as shown in Figure 2c. According to the knowledge of amplitude‐phase joint coding metasurface reported in our previous work,^[^ ^47^ ^]^ the reflection amplitude and phase of the unit element can be independently controlled by adjusting the variable resistor of PIN diodes. Figures 2d and 2e show the simulation results of the reflection amplitude and phase of the unit element with *R*_d_ *_x_* and *R*_d_ *_y_* at 10 GHz, under *x*‐ and *y*‐polarized incidences, respectively. The results show that the amplitude of *x* (or *y*) polarized reflected wave can be continuously controlled from 1 to 0 as *R*_d_ *_x_* (or *R*_d_ *_y_*) increases from 1 to 30 Ω or decreases from 10 000 to 30 Ω, but the phase difference of reflected waves in these two regions keeps about 180°. Therefore, the amplitude and phase of both *x*‐ and *y*‐polarized reflected waves can be independently controlled by the PIN diodes loaded along the *x*‐ and *y*‐directions, respectively. In addition, the proposed unit element also has broadband characteristics and can operate in the frequency of 9–11 GHz (see Figure S2, Supporting Information).

When a 45°‐polarized wave illuminates to the metasurface, the polarization angle of its reflected wave can be arbitrarily customized by controlling the amplitude and phase of *x*‐ and *y*‐polarization components. Figure 2f–i shows the simulated results of 45°, *x*, *y*, and −45° polarized reflection waves, where the metasurface is composed of 20 × 20 elements with a total size of 284 × 284 mm^2^. When the resistances of PIN diodes along *x*‐ and *y*‐directions are set to *R*_d_ *_x_* = *R*_d_ *_y_* = 10 000 Ω, both *x*‐ and *y*‐polarization components are efficiently reflected and have the same amplitude and phase, so the reflected wave has the same polarization as the incident wave, that is, 45° polarization, as shown in Figure 2g. When resistances of PIN diodes along *x*‐ and *y*‐directions are set to *R*_d_ *_x_* = 10 000 Ω and *R*_d_ *_y_* = 30 Ω, respectively, only the *x* polarization component is efficiently reflected, while the *y* polarization component is completely absorbed, so the reflected wave will be *x* polarization, as shown in Figure 2h. Similarly, when resistances of PIN diodes along *x*‐ and *y*‐directions are set to *R*_d_ *_x_* = 30 Ω and *R*_d_ *_y_* = 10 000 Ω, respectively, only the *y* polarization component is efficiently reflected, while the *x* polarization component is completely absorbed, the reflected waves will be *y* polarization, as shown in Figure 2i. However, when resistances of PIN diodes along *x*‐ and *y*‐directions are set to *R*_d_ *_x_* = 10 000 Ω and *R*_d_ *_y_* = 1 Ω, respectively, both the *x*‐ and *y*‐polarization components are efficiently reflected with the same reflection amplitude, but have a phase difference of 180°, so the reflected wave will be cross‐polarization of the incident wave, that is, −45° polarization, as shown in Figure 2i. It is worth noting that although only four special cases are demonstrated, the reflected wave with arbitrary polarization angle can be achieved by accurately controlling the amplitude and phase of *x*‐ and *y*‐reflection components. In this work, 45°, *x*, *y*, and −45° polarization channels are used for the encryption scheme, which are encoded as polarization codes of “00,” “01,” “10,” and “11,” respectively. In addition, when a left‐handed circularly polarized wave illuminates to the metasurface, the polarization ellipticity of its reflected wave can also be arbitrarily customized by controlling the amplitude and phase of *x*‐ and *y*‐polarization components (see Figure S3, Supporting Information, for details).

### 3.2 Measurement Results

**Figure 3:**
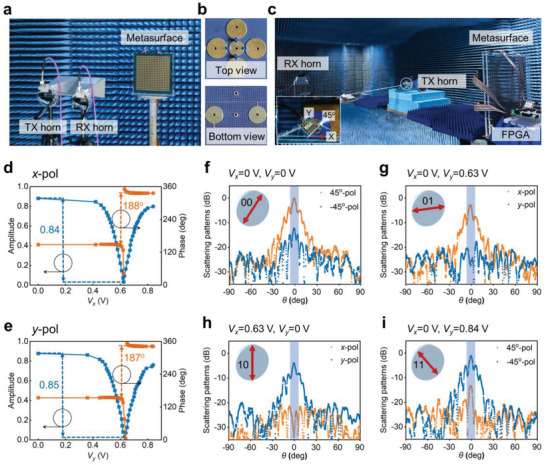
Experimental results of the PoM metasurface. a) Experimental setup for measuring the reflection coefficients in an anechoic chamber. b) Top view of one unit element. c) Bottom view of one unit element. d,e) The measured reflection amplitudes and phases of the metasurface applied by different bias voltages under the illumination of d) *x*‐polarized and e) *y*‐polarized incident waves at 10 GHz. f–i) The measured 2D far‐field radiation patterns for four linear polarization states: f) 45° polarization, g) *x* polarization, h) *y* polarization, and i) −45° polarization.

The programmable PoM information metasurface is fabricated and measured in an anechoic chamber, as shown in **Figure** **3**, where the metasurface is composed of 20 × 20 elements with a total size of 284 × 284 mm^2^, and the PIN diodes along horizontal and vertical directions are independently controlled by two bias voltages. Figure 3a shows the experimental setup for measuring the reflection amplitude and phase, in which two rectangular horn antennas are placed 1.5 m away from the metasurface for emitting and receiving the signals, respectively. The photograph of one of the units is illustrated in Figure 3b, in which the top view shows the unit element loaded with PIN diodes and the bottom view shows the structure of feeder lines. Figure 3d shows the measured results of the amplitude and phase of *x*‐polarized reflection wave at 10 GHz. When the bias voltage *V_x_* continuously changes from 0 to 0.84 V, the amplitude of reflected wave can be continuously adjusted from 0.02 to 0.87, while the phase can be independently switched between two phases with a difference of about 188°. The similar result also can be obtained for *y*‐polarized wave, as shown in Figure 3e. The measured results are in good agreement with the simulations shown in Figure 2c,d. In addition, the broadband characteristics of the metasurface also have been validated by measurements (see Figure S4, Supporting Information, for details).

The far‐field radiation patterns of the programmable PoM information metasurface are measured in a standard microwave anechoic chamber to verify the performance of polarization modulation, as shown in Figure 3c. The metasurface is placed on a rotating platform, and an x‐band metamaterial lens antenna^[^ ^48^ ^]^ is placed 80 cm away from the metasurface to generate a 45°‐polarized incident plane wave. An X‐band standard rectangular horn antenna is fixed in another side of the anechoic chamber as a receiver, where the distance between the receiving horn antenna and the rotating platform is about 10 m. The measured far‐field radiation patterns of different linearly polarized reflected waves under different bias voltages are shown in Figure 3f–i. When bias voltages of PIN diodes along *x*‐ and *y*‐directions are set to *V_x_*/*V_y_* = 0 V/0 V, 0 V/0.63 V, 0.63 V/0 V, and 0 V/0.84 V, the reflected wave will be 45°, *x*, *y*, and −45° polarization, respectively. In addition, the measured far‐field radiation patterns of different polarized reflected waves under a circularly polarized incident wave can be seen in Figure S5, Supporting Information.

## 4 Implementation of the PoM‐Encrypted Wireless Communication System

**Figure 4:**
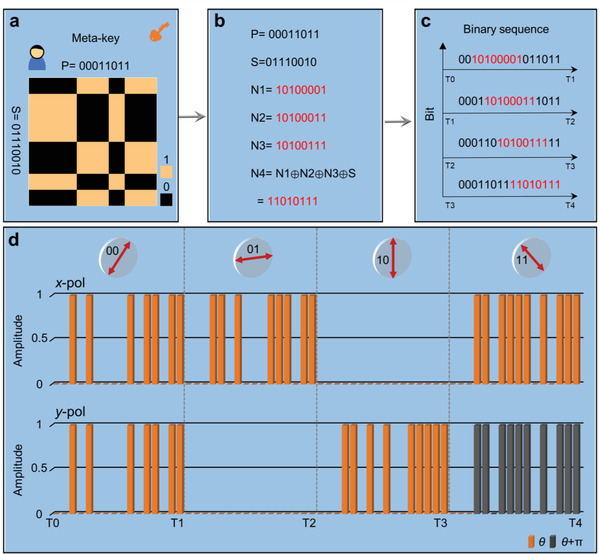
Encryption process of the PoM‐encrypted wireless communication scheme. a–c) The encryption process with four binary sequences as the meta‐key. d) Two orthogonal input voltage signals of the *x*‐ and *y*‐polarized waves under different linear‐polarization channels, respectively.

The block diagram of the meta‐key encryption scheme based on the PoM metasurface is demonstrated in **Figure** **4**. The meta‐key is composed of two 8‐bit binary numbers of P and S, as shown in Figure 4a, in which the *M*th row and *N*th column element of the meta‐key matrix are achieved by exclusive OR (XOR) operation of the *M*th element of S and *N*th element of P. Figure 4b shows the coding rule of P and S. In this encryption scheme, P is encoded as 00011011, representing the information of polarization channels, in which codes “00,” “01,” “10,” and “11” are corresponding to 45°, *x*, *y*, and −45° polarization channels, respectively. S could be any binary sequence, which is encoded as an 8‐bit binary number of 01110010 in this case. In order to avoid the information of S being intercepted easily, we introduce four other 8‐bit binary numbers of N1, N2, N3, and N4, in which N1, N2, and N3 can be random 8‐bit binary numbers, while N4 = S ⊕ N1⊕N2⊕N3 (“⊕” is the XOR symbol). To further increase the security of the meta‐key, we insert N1, N2, N3, and N4 into the sequence of P, respectively, to generate four 16‐bit binary sequences, in which N1, N2, N3, and N4 are inserted behind the polarization channel codes of “00,” “01,” “10,” and “11,” respectively, and then these four 16‐bit binary numbers are circularly sent in turn by using the corresponding polarization channel through the PoM metasurface, as shown in Figure 4c. Taking the *x* polarization channel as an example, N2 = 10100011 is inserted behind the code “01” of P to generate a new 16‐bit binary number of 0001101000111011, which is sent by using the *x* polarization channel during the time period of T1–T2. For this 16‐bit binary number, we redefine the meanings of codes “0” and “1,” which represent the low and high‐level signals, respectively, corresponding to the amplitude of EM wave in each polarization channel, that is low reflection represents code “0” and high reflection represents code “1.” Figure 4d shows the required amplitude and phase distributions of *x*‐ and *y*‐polarized waves with respect to the corresponding coding sequence in each polarization channel during the time period of T0–T4. It is worth mentioning that the height of the cuboid represents the amplitude of EM waves, and the orange and black colors indicate that their phases are *θ* and *θ* + *π*, respectively. Taking the 45° polarization channel as an example, the amplitude and phase of *x*‐ and *y*‐polarized waves should be the same to ensure the EM wave is 45° polarization, and then the amplitude of 45°‐polarized EM wave is further modulated to vary with time, generating the required coding sequence of 0010100001011011, as shown in the time period of T0–T1 in Figure 4d. The coding sequences in other polarization channels are realized by using the similar method, which are also shown in Figure 4d.

**Figure 5:**
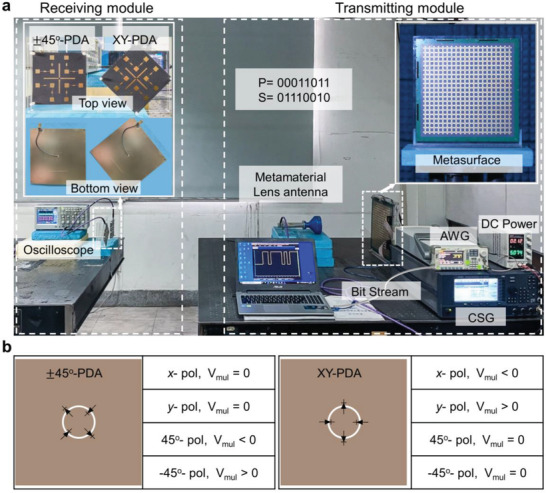
Experimental setup of the PoM wireless communication system. a) The testbed of the system, photographs of the metasurface, and photographs of the top view and bottom view of the ±45°‐PDA and XY‐PDA, respectively. b) The output voltages of two PDAs at different linear polarization states.

A wireless communication system was set up based on the proposed programmable PoM information metasurface to implement the abovementioned encryption scheme in an indoor scenario, as shown in **Figure** **5**a. It consists of a transmitting module and a receiving module, in which the transmitting module is composed of a metamaterial lens antenna, a PoM metasurface, a carrier signal generator (Keysight E8267D), a spectrum analyzer (Keysight N9040B), an arbitrary waveform generator (AWG) (Rigol DG5101), and a DC stabilized power supply, and the receiving module is composed of an oscilloscope and two PDAs. In the transmitting module, the metamaterial lens antenna is placed 35 cm in front of the metasurface to generate a 45°‐polarized incident plane wave, which can be regarded as the carrier wave with a working frequency of *f_c_*; the AWG and DC stabilized power supply are connected to the metasurface for providing the bias voltage to PIN diodes. It is worth mentioning that two AWGs were required to provide real‐time modulated row‐ and column‐controlled bias voltages for metasurface, respectively, but since we only have one AWG, a DC stabilized power supply is used to take place of another AWG in the experiment. The time‐varying bias voltage is supplied to the metasurface through AWG to realize the real‐time modulation of polarization and amplitude of the reflected wave, so as to obtain the desired polarization channel and its corresponding coding sequence. In receiving module, a pair of PDAs are placed 160 cm away from the PoM metasurface to receive the signal, which are connected to a dual channel oscilloscope. The photographs of PDAs are displayed in the upper left corner of Figure 5a, in which two PDAs are placed with a related rotation angle of 45°, named as ±45°‐PDA and XY‐PDA, respectively. On the back of the PDA, four zero‐bias Schottky diodes (SMS7621‐040LF) are loaded on the slot ring to realize the double‐balanced RF multiplier. For more details about the design of PDA, please see Figure S6, Supporting Information. When the PDAs receive signals, the polarization state of the signal can be first identified according to the output voltage of two PDAs, as shown in Figure 5b. To be specific, if the output voltages of ±45°‐PDA and XY‐PDA are a large negative (or positive) value and zero, respectively, the signal will be 45° (or −45°) polarization, while if the output voltages of ±45°‐PDA and XY‐PDA are zero and a large negative (or positive) value, respectively, the signal will be *x* (or *y*) polarization.^[^ ^49^ ^]^ Once the polarization state of the signal is determined, then the corresponding coding sequence of “0” and “1” in each polarization channel can be further obtained through the low‐ and high‐level output voltages detected by the oscilloscope.

**Figure 6:**
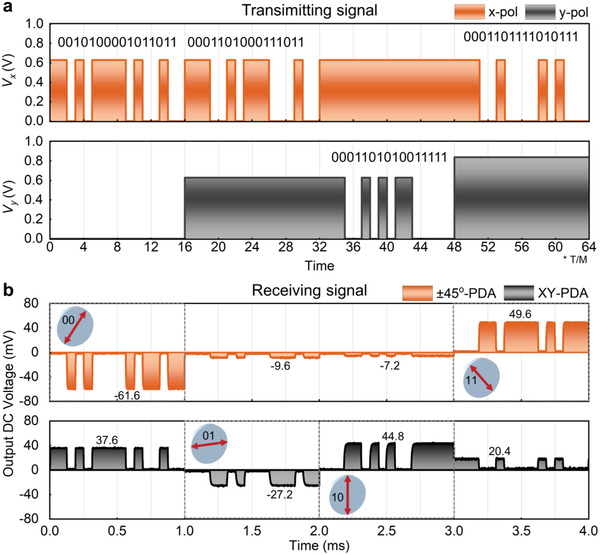
Waveforms of the transmitting signals and receiving signals. a) The time‐varying bias voltages of AWG module used to control the PoM metasurface. b) The measured output DC voltages of two PDAs under the control of AWG.

**Figure** **6**a illustrates the time‐varying bias voltage sequences of AWG and DC stabilized power supply in the experiment to drive the PoM metasurface, in which 0.63 V corresponds to low reflection, and both 0 and 0.84 V correspond to high reflection, but their phase difference is 180°. The time interval for each code is *T*/*M* = 0.0625 ms (*T* = 4 ms, *M* = 64), so the data rate during transmission is 16 kHz, and the maximum data rate mainly depends on the modulation rate of the AWG and the switching rate of the PIN diode.^[^ ^27^ ^]^ It is worth mentioning that one of the bias voltages (*V_x_* or *V_y_*) remains unchanged in every polarization channel, which is because it is provided by the DC stabilized power supply in the experiment. Figure 6b shows the output voltages of ±45°‐PDA and XY‐PDA measured by oscilloscope in four different time periods. The polarization of the signal can first be identified by the relationship between the output voltages of ±45°‐PDA and XY‐PDA, according to the rule shown in Figure 5b. Therefore, we can know that the signals shown in Figure 6b are 45°, *x*, *y*, and −45° polarizations from the left to right. It is worth mentioning that the signal received by XY‐PDA is much larger than zero in 45° (“00”) and −45° (“11”) polarization channels, which should be close to zero as discussed in Figure 5b. The reason for this result is that the bias voltage for controlling *y*‐polarized wave in these two cases is provided by a DC stabilized power supply, which is set to a fixed voltage of 0 and 0.84 V, respectively, so the *y* polarization component cannot be eliminated. On the other hand, the signal level received by XY‐PDA in −45° polarization channel is slightly lower than that in 45° polarization channel, which is because the reflected amplitude of *y*‐polarized wave under the bias voltage of 0.84 V is a little bit smaller than that of 0 V. This problem can be solved by using another AWG to take place of the DC stabilized power supply. After knowing the polarization channels, according to the encryption protocol displayed in Figure 4, we can extract N1, N2, N3, and N4 after codes “00” in the 45° polarization channel, “01” in the *x* polarization channel, “10” in *y* polarization channel, and “11” in −45° polarization channel, respectively. In addition, we can identify whether the extraction results are correct by checking whether the remaining 8‐bit binary numbers in all channels are P = 00011011. Then, S can be obtained by bitwise XOR operation of N1, N2, N3, and N4. Therefore, according to the above rules, the binary coding streams of 0010100001011011, 0001101000111011, 0001101010011111, and 0001101111010111 in four different polarization channels can be first read from the measured results shown in Figure 6b, respectively, and then N1 = 10100001, N2 = 10100011, N3 = 10100111, and N4 = 11010111 can be further extracted to obtain the final P = 00011011 and S = N1⊕N2⊕N3⊕N4 = 01110010. Then, the matrix of meta‐key can be obtained by performing the XOR operation of P and S, as shown in Figure 4a. Finally, the encrypted lynx image can be recovered by using the meta‐key. In addition, we further investigate the fault‐tolerant performance of meta‐key. The results show that the target image can also be roughly observed if there are only one‐ or two‐bits error in the receiving process of N1–N4, but the resolution of image will become lower and lower as the bit error rate increases, and the image is completely unrecognizable when the number of error code reaches four (see Figure S7, Supporting Information).

## 5 Conclusion

We have proposed a programmable PoM information metasurface and applied it to the encryption wireless communications by introducing a meta‐key. In this scheme, the meta‐key is sent to the user independently in real‐time, it can be dynamically adjusted for the different messages to enhance security. A wireless communication system, including transmitting and receiving modules, has been built to demonstrate the feasibility of the encryption scheme. The approach offers a low‐cost solution for implementing PoM‐encrypted wireless communications network, which does not need antenna arrays, filters, and mixers. The work provides a new concept for information security transmission based on the programmable metasurface, which may find great potential in the next generation of information encryption.

## Conflict of Interest

The authors declare no conflict of interest.

## Supporting information

Supporting Information

## Data Availability

The data that support the findings of this study are available from the corresponding author upon reasonable request.

## References

[1] N. Yu , P. Genevet , M. A. Kats , F. Aieta , J.‐P. Tetienne , F. Capasso , Z. Gaburro , Science 2011, 334, 333. 21885733 10.1126/science.1210713

[2] Y. Yang , I. I. Kravchenko , D. P. Briggs , J. Valentine , Nat. Commun. 2014, 5, 5753. 25511508 10.1038/ncomms6753

[3] A. Arbabi , Y. Horie , M. Bagheri , A. Faraon , Nat. Nanotechnol. 2015, 10, 937. 26322944 10.1038/nnano.2015.186

[4] X. Zang , F. Dong , F. Yue , C. Zhang , L. Xu , Z. Song , M. Chen , P.‐Y. Chen , G. S. Buller , Y. Zhu , S. Zhuang , W. Chu , S. Zhang , X. Chen , Adv. Mater. 2018, 30, 1707499. 10.1002/adma.201707499 29603423

[5] G. Minatti , F. Caminita , E. Martini , M. Sabbadini , S. Maci , IEEE Trans. Antennas Propag. 2016, 64, 3907.

[6] H. L. Zhu , S. W. Cheung , K. L. Chung , T. I. Yuk , IEEE Trans. Antennas Propag. 2013, 61, 4615.

[7] X. Gao , X. Han , W.‐P. Cao , H. O. Li , H. F. Ma , T. J. Cui , IEEE Trans. Antennas Propag. 2015, 63, 3522.

[8] P. Yang , R. Dang , L. Li , IEEE Trans. Antennas Propag. 2022, 10.1109/TAP.2022.3178803.

[9] Q. Song , A. Baroni , R. Sawant , P. Ni , V. Brandli , S. Chenot , S. Vézian , B. Damilano , P. de Mierry , S. Khadir , P. Ferrand , P. Genevet , Nat. Commun. 2020, 11, 2651. 32461637 10.1038/s41467-020-16437-9 PMC7253437

[10] E. Maguid , I. Yulevich , M. Yannai , V. Kleiner , M. L. Brongersma , E. Hasman , Light: Sci. Appl. 2017, 6, e17027. 30167279 10.1038/lsa.2017.27 PMC6062311

[11] N. Mao , G. Zhang , Y. Tang , Y. Li , Z. Hu , X. Zhang , K. Li , K. Cheah , G. Li , Proc. Natl. Acad. Sci. U. S. A. 2022, 119, e2204418119. 35617434 10.1073/pnas.2204418119 PMC9295796

[12] J. W. Wu , Z. X. Wang , Z. Q. Fang , J. C. Liang , X. Fu , J. F. Liu , H. T. Wu , D. Bao , L. Miao , X. Y. Zhou , Q. Cheng , T. J. Cui , Adv. Funct. Mater. 2020, 30, 2004144.

[13] Y. Yuan , S. Sun , Y. Chen , K. Zhang , X. Ding , B. Ratni , Q. Wu , S. N. Burokur , C. Qiu , Adv. Sci. 2020, 7, 2001437. 10.1002/advs.202001437 PMC7509705 32999848

[14] Y. Yuan , K. Zhang , B. Ratni , Q. Song , X. Ding , Q. Wu , S. N. Burokur , P. Genevet , Nat. Commun. 2020, 11, 4186. 32826879 10.1038/s41467-020-17773-6 PMC7442839

[15] Y. Hu , X. Wang , X. Luo , X. Ou , L. Li , Y. Chen , P. Yang , S. Wang , H. Duan , Nanophotonics 2020, 9, 3755.

[16] L. Li , T. J. Cui , W. Ji , S. Liu , J. Ding , X. Wan , Y. B. Li , M. Jiang , C.‐W. Qiu , S. Zhang , Nat. Commun. 2017, 8, 197. 28775295 10.1038/s41467-017-00164-9 PMC5543116

[17] J. Li , P. Yu , S. Zhang , N. Liu , Nat. Commun. 2020, 11, 3574. 32681122 10.1038/s41467-020-17390-3 PMC7367846

[18] S. Venkatesh , X. Lu , H. Saeidi , K. Sengupta , Nat. Electron. 2020, 3, 785.

[19] M. A. ElMossallamy , H. Zhang , L. Song , K. G. Seddik , Z. Han , G. Y. Li , IEEE Trans. Cognit. Commun. Networking 2020, 6, 990.

[20] Y. Liu , X. Liu , X. Mu , T. Hou , J. Xu , M. Di Renzo , N. Al‐Dhahir , IEEE Commun. Surv. Tutorials 2021, 23, 1546.

[21] J. Tao , Q. You , Z. Li , M. Luo , Z. Liu , Y. Qiu , Y. Yang , Y. Zeng , Z. He , X. Xiao , G. Zheng , S. Yu , Adv. Mater. 2022, 34, 2106080. 10.1002/adma.202106080 34825747

[22] C. Qian , B. Zheng , Y. Shen , L. Jing , E. Li , L. Shen , H. Chen , Nat. Photonics 2020, 14, 383.

[23] J. W. You , Q. Ma , Z. Lan , Q. Xiao , N. C. Panoiu , T. J. Cui , Nat. Commun. 2021, 12, 5468. 34526488 10.1038/s41467-021-25835-6 PMC8443663

[24] Q. Ma , Q. R. Hong , G. D. Bai , H. B. Jing , R. Y. Wu , L. Bao , Q. Cheng , T. J. Cui , Phys. Rev. Appl. 2020, 13, 021003.

[25] X. Gao , W. L. Yang , H. F. Ma , Q. Cheng , X. H. Yu , T. J. Cui , IEEE Trans. Antennas Propag. 2018, 66, 6086.

[26] Z. Wu , Y. Ra'di , A. Grbic , Phys. Rev. X 2019, 9, 011036.

[27] C. X. Huang , J. Zhang , Q. Cheng , T. J. Cui , Adv. Funct. Mater. 2021, 31, 2103379.

[28] M. Cerveny , K. L. Ford , A. Tennant , IEEE Trans. Antennas Propag. 2021, 69, 1483.

[29] P. Yu , J. Li , N. Liu , Nano Lett. 2021, 21, 6690. 34286586 10.1021/acs.nanolett.1c02318 PMC8361430

[30] Y. Hu , X. Ou , T. Zeng , J. Lai , J. Zhang , X. Li , X. Luo , L. Li , F. Fan , H. Duan , Nano Lett. 2021, 21, 4554. 34047184 10.1021/acs.nanolett.1c00104

[31] T. Badloe , I. Kim , Y. Kim , J. Kim , J. Rho , Adv. Sci. 2021, 8, 2102646. 10.1002/advs.202102646 PMC8564427 34486242

[32] J. Y. Dai , J. Zhao , Q. Cheng , T. J. Cui , Light: Sci. Appl. 2018, 7, 90. 30479756 10.1038/s41377-018-0092-z PMC6249241

[33] M. Z. Chen , W. Tang , J. Y. Dai , J. C. Ke , L. Zhang , C. Zhang , J. Yang , L. Li , Q. Cheng , S. Jin , T. J. Cui , Natl. Sci. Rev. 2022, 9, nwab134. 35079409 10.1093/nsr/nwab134 PMC8783670

[34] J. Yang , J. C. Ke , W. K. Cao , M. Z. Chen , Q. Cheng , V. Galdi , T. J. Cui , Adv. Opt. Mater. 2021, 9, 2101043.

[35] Q. Hu , K. Chen , N. Zhang , J. Zhao , T. Jiang , J. Zhao , Y. Feng , Adv. Opt. Mater. 2022, 10, 2101915.

[36] J. C. Ke , J. Y. Dai , M. Z. Chen , L. Wang , C. Zhang , W. Tang , J. Yang , W. Liu , X. Li , Y. Lu , Q. Cheng , S. Jin , T. J. Cui , Small Struct. 2021, 2, 2000060.

[37] P. Zheng , Q. Dai , Z. Li , Z. Ye , J. Xiong , H.‐C. Liu , G. Zheng , S. Zhang , Sci. Adv. 2021, 7, eabg0363. 34020956 10.1126/sciadv.abg0363 PMC8139587

[38] J. Li , S. Kamin , G. Zheng , F. Neubrech , S. Zhang , N. Liu , Sci. Adv. 2018, 4, eaar6768. 29922715 10.1126/sciadv.aar6768 PMC6003725

[39] Q. Xiao , Q. Ma , T. Yan , L. W. Wu , C. Liu , Z. X. Wang , X. Wan , Q. Cheng , T. J. Cui , Adv. Opt. Mater. 2021, 9, 2002155.

[40] L. W. Wu , Q. Xiao , Y. Gou , R. Y. Wu , P. Xu , Y. M. Qing , Z. X. Wang , L. Bao , H. F. Ma , T. J. Cui , Adv. Opt. Mater. 2022, 10, 2102657.

[41] T. J. Cui , M. Q. Qi , X. Wan , J. Zhao , Q. Cheng , Light: Sci. Appl. 2014, 3, e218.

[42] L. Zhang , M. Z. Chen , W. Tang , J. Y. Dai , L. Miao , X. Y. Zhou , S. Jin , Q. Cheng , T. J. Cui , Nat. Electron. 2021, 4, 218.

[43] J. Y. Dai , W. K. Tang , J. Zhao , X. Li , Q. Cheng , J. C. Ke , M. Z. Chen , S. Jin , T. J. Cui , Adv. Mater. Technol. 2019, 4, 1900044.

[44] X. Wan , Q. Zhang , T. Y. Chen , L. Zhang , W. Xu , H. Huang , C. K. Xiao , Q. Xiao , T. J. Cui , Light: Sci. Appl. 2019, 8, 60. 31645910 10.1038/s41377-019-0169-3 PMC6804601

[45] X. G. Zhang , Y. L. Sun , B. Zhu , W. X. Jiang , Q. Yu , H. W. Tian , C.‐W. Qiu , Z. Zhang , T. J. Cui , Light: Sci. Appl. 2022, 11, 126. 35513383 10.1038/s41377-022-00817-5 PMC9072331

[46] Q. Ma , W. Gao , Q. Xiao , L. Ding , T. Gao , Y. Zhou , X. Gao , T. Yan , C. Liu , Z. Gu , X. Kong , Q. H. Abbasi , L. Li , C.‐W. Qiu , Y. Li , T. J. Cui , eLight 2022, 2, 11.

[47] H. L. Wang , Y. K. Zhang , T. Y. Zhang , H. F. Ma , T. J. Cui , ACS Appl. Mater. Interfaces 2022, 14, 29431. 35709434 10.1021/acsami.2c05907

[48] X. Chen , H. F. Ma , X. Y. Zou , W. X. Jiang , T. J. Cui , J. Appl. Phys. 2011, 110, 044904.

[49] M. A. Hossain , E. Nishiyama , M. Aikawa , I. Toyoda , PIER C 2013, 34, 53.

