# Supplementary material for: A Polarization‐Modulated Information Metasurface for Encryption Wireless Communications
Source: Adv Sci (Weinh). 2022 Oct 17;9(34):2204333. doi: 10.1002/advs.202204333 (PMC9731708; doi:10.1002/advs.202204333)
Supplement: Supplementary file 1 — Supporting Information [file ADVS-9-2204333-s001.pdf]

ADVANCED SCIENCE
Open Access

Supporting Information

for *Adv. Sci.*, DOI 10.1002/advs.202204333

A Polarization-Modulated Information Metasurface for Encryption Wireless Communications

Hai Lin Wang, Hui Feng Ma and Tie Jun Cui**

Supporting Information for

A polarization-modulated information metasurface for encryption wireless communications

Hai Lin Wang, Hui Feng Ma and Tie Jun Cui**

[1]State Key Laboratory of Millimeter Waves, School of Information Science and Engineering, Southeast University, Nanjing 210096, China
[2]Institute of Electromagnetic Space, Southeast University, Nanjing, 210096, China

*Corresponding author. Email: hfma@seu.edu.cn, tjcui@seu.edu.cn.

The Supporting Information includes:

- S1. Encryption and decryption processes
- S2. Simulated results of amplitude and phase responses of PoM metasurface
- S3. Simulated 2D far-field radiation reflected patterns
- S4. Measured results of amplitude and phase responses of PoM metasurface
- S5. Measured 2D far-field radiation reflected patterns
- S6. Polarization discrimination antenna (PDA) and its simulation results
- S7. Fault-tolerant performance of meta-key.

S1. Encryption and decryption processes

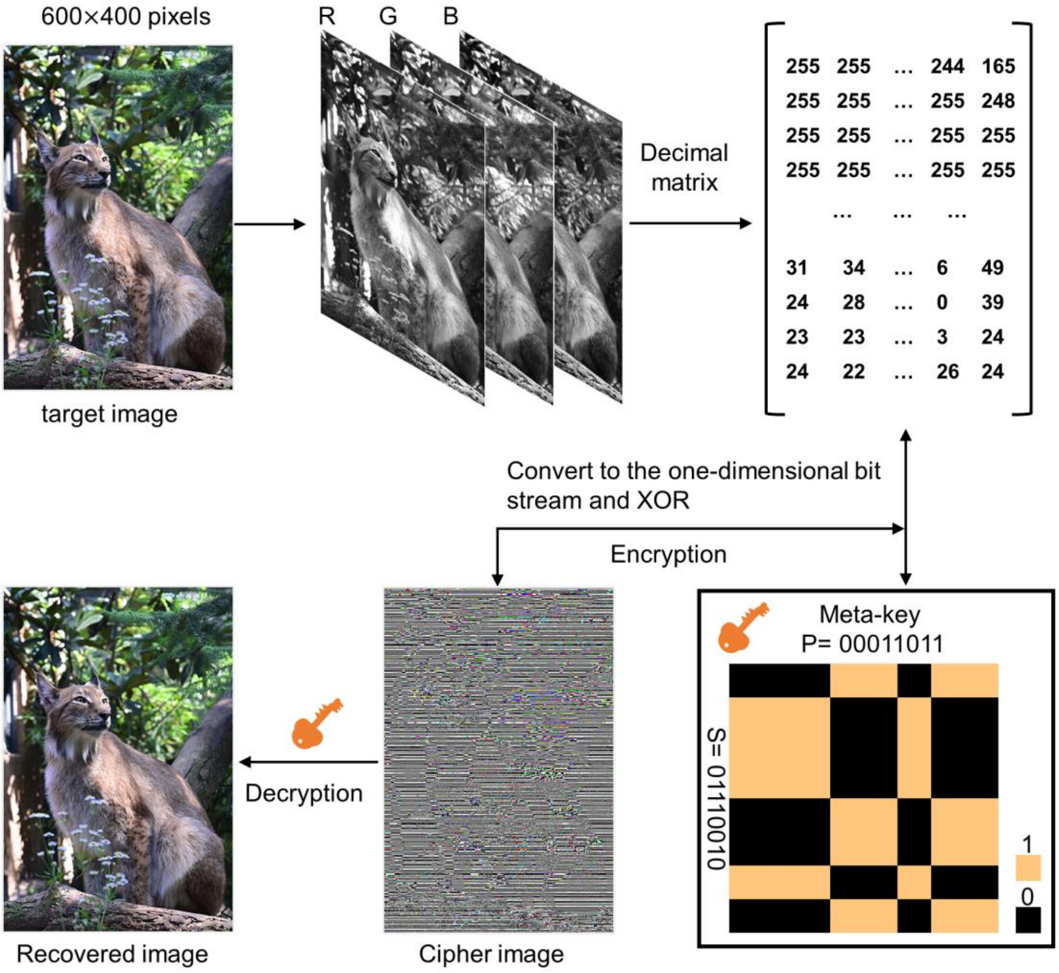

Figure S1. Scheme of the encryption process with modulated signals as the meta-key and decryption process with demodulated signals as the meta-key. Photo credit: Hai Lin Wang, Southeast University.

Figure S1 shows the scheme of encryption and decryption processes for an image by adopting a meta-key. In the transmitting end, the target image (i.e., lynx) can be decomposed to grayscale images of RGB and encoded as a matrix of binary numbers ($T^{m\times n}$), and then the matrix is encrypted by using the meta-key following the principle of simple XOR operation to generate a cipher image. If the user only receives information of cipher image, he/she cannot know the true target image. Hence, the meta-key should also be encrypted and sent to the user, which is focus of this work and can be achieved by using the proposed programmable PoM information metasurface. Once the user gets the correct meta-key, the real information of target image can be obtained by the XOR operation of the meta-key and encrypted matrix of

cipher image.

S2. Simulated results of amplitude and phase responses of PoM metasurface

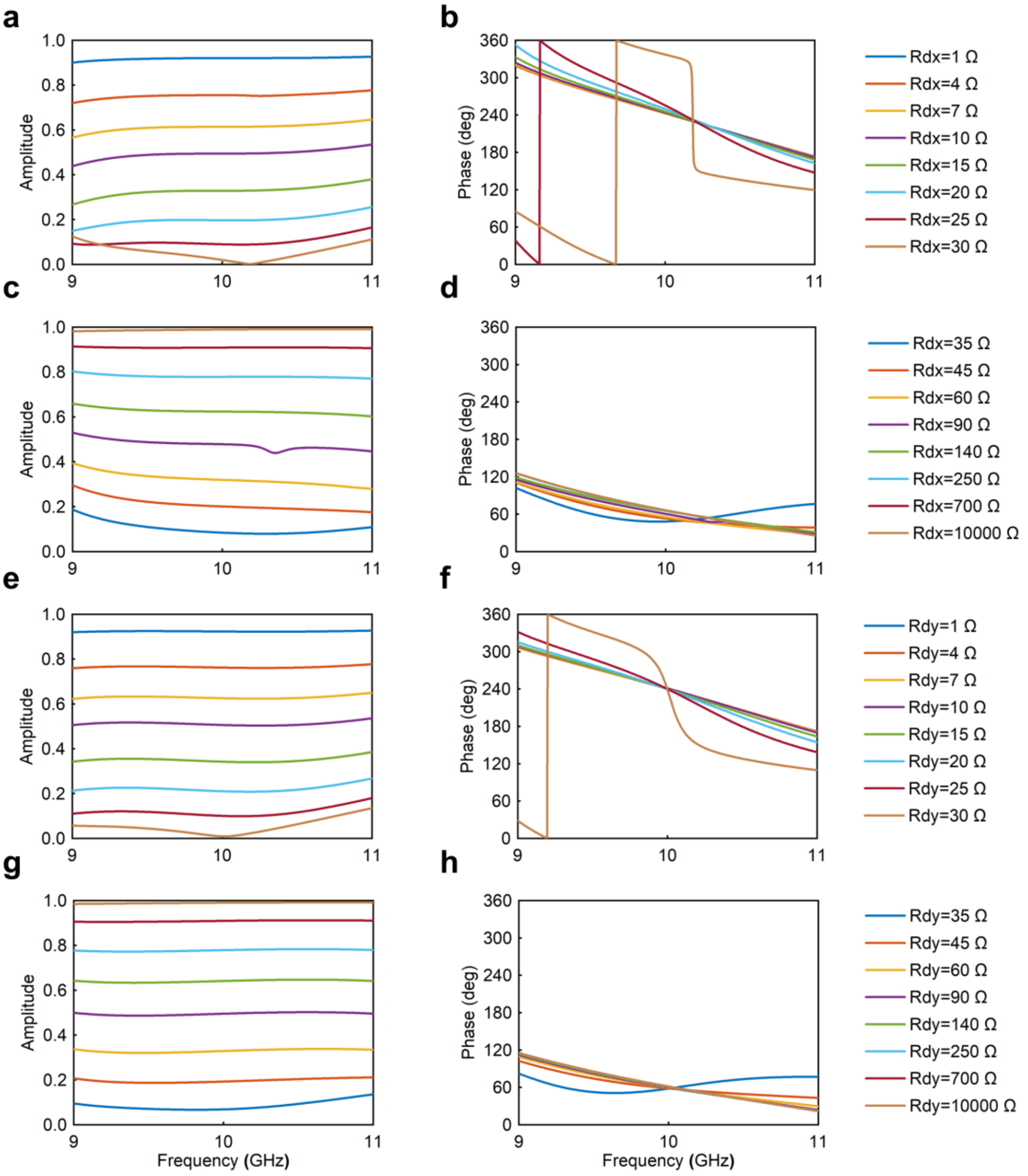

Figure S2. The simulated reflection amplitude and phase of unit element varying with Rdx and Rdy in a wide band of 9-11 GHz. a) Simulated reflection amplitude of *x*-polarized wave with Rdx=1 to 30 Ω. b) Simulated reflection phase of *x*-polarized wave with Rdx=1 to 30 Ω. c) Simulated reflection amplitude of *x*-polarized wave with Rdx=35 to 10000 Ω. d) Simulated reflection phase of *x*-polarized wave with Rdx=35 to 10000 Ω. e) Simulated reflection amplitude of *y*-polarized wave with Rdy=1 to 30 Ω. f) Simulated reflection phase of *y*-polarized wave with Rdy=35 to 10000 Ω. g) Simulated reflection amplitude of *y*-polarized wave with Rdy=1 to 30 Ω. h) Simulated reflection phase of *y*-polarized wave with Rdy=35 to 10000 Ω.

Figures S2a-d demonstrate the simulated reflection coefficient (S11) of the unit

element varying with Rdx and Rdy in the frequency band of 9 to 11 GHz. When Rdx is increased from 1 to 30 Ω, the reflection amplitude keeps decreasing from about 0.9 to 0 but the reflection phase remains basically unchanged, as shown in Figures S2a and S2b. However, when Rdx is increased continuously from 35 to 10000 Ω, the reflection amplitude keeps increasing from about 0.1 to 1 but the reflection phase also remains basically unchanged, as shown in Figures S2c and S2d. It is worth mentioning that there are slight disturbances in the phase responses at the low-frequency band as Rdx=25 and 30 Ω and the high-frequency band as Rdy=35 Ω, respectively, but because these unit elements are all almost working in absorption state with low reflection amplitude, the phase disturbances have little influence with the final results of the metasurface. The results show that the reflection amplitude can be continuously controlled from 1 to 0 when Rd changes in both regions of 1 to 30 Ω and 35 to 10000 Ω, while the phase difference of reflected waves remains about 180° in these two regions. Hence, the amplitude and phase of *x*-polarized reflected waves can be independently controlled by changing the Rdx of the PIN diode. The similar results can be achieved for y-polarized wave, as shown in Figures S2e-h.

S3. Simulated 2D far-field radiation reflected patterns for four different polarization states

Figures S3f-i show the simulated results of RCP, *x* polarization, *y* polarization, and LCP. When the resistances of PIN diodes along *x* and *y* directions are set to R_{dx}=R_{dy}=10000 Ω, both *x* and *y* polarization components are efficiently reflected and have the same amplitude and phase, so the reflected wave has the right-handed circularly polarization as the left-handed circular polarized incident wave, as shown in Figure S3a. When resistances of PIN diodes along *x* and *y* directions are set to R_{dx}=10000 Ω and R_{dy}=30 Ω, respectively, only the *x* polarization component is efficiently reflected, while the *y* polarization component is completely absorbed, so the reflected wave will be *x* polarization, as shown in Figure S3b. Similarly, when resistances of PIN diodes along *x* and *y* directions are set to R_{dx}=30 Ω and R_{dy}=10000 Ω, respectively, only the *y* polarization component is efficiently reflected, while the *x*

polarization component is completely absorbed, and the reflected waves will be *y* polarization, as shown in Figure S3c. However, when resistances of PIN diodes along *x* and *y* directions are set to R_{dx}=10000 Ω and R_{dy}=1 Ω, respectively, both the *x* and *y* polarization components are efficiently reflected with the same reflection amplitude, but have a phase difference of 180°, so the reflected wave will be left-handed circularly polarization of incident wave, as shown in Figure S3d. It is worth noting that although only four special cases are demonstrated, the reflected wave with arbitrary polarization ellipticity can be achieved by accurately controlling the amplitude and phase of the *x* and *y* reflection components.

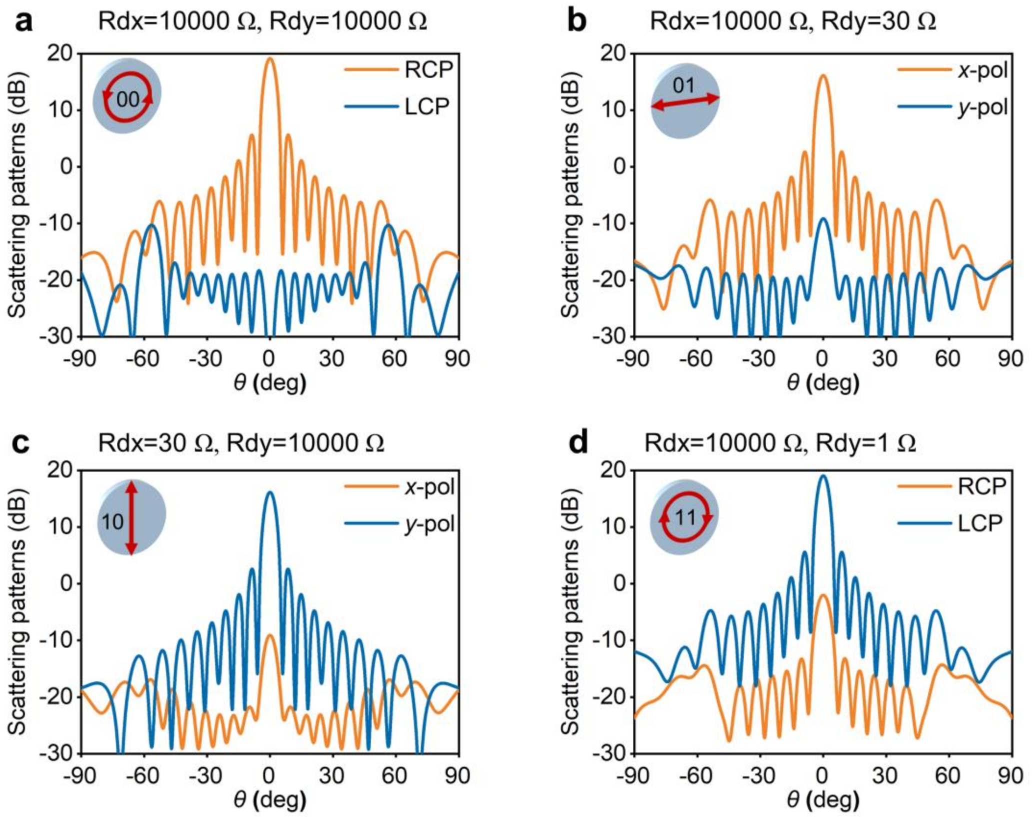

Figure S3. Measured 2D far-field radiation reflected patterns of a) right-handed circular polarization (RCP), b) *x* polarization, c) *y* polarization, and d) left-handed circular polarization (LCP).

S4. Measured results of amplitude and phase responses of PoM metasurface

Figures S4a and 4b illustrate the measured amplitude and phase responses of S11 under the *x*-polarized incidence as the bias voltage increases from 0 to 0.63 V, where the voltage accuracy can achieve 0.01 V. The results show that the reflection

amplitude continuously decreases in a wide band of 9 to 11 GHz, but the reflection phase almost remains unchanged. Figure S4c and 4d illustrate the measured amplitude and phase responses of S11 under the *x*-polarized incidence as the bias voltage is further increased from 0.64 to 0.83 V, which show that the reflection amplitude continuously increases in a wide band of 9 to 11 GHz, and the reflection phase almost remains unchanged. The similar measurement results for *y*-polarized incidence can be achieved, as shown in Figures S4e-h.

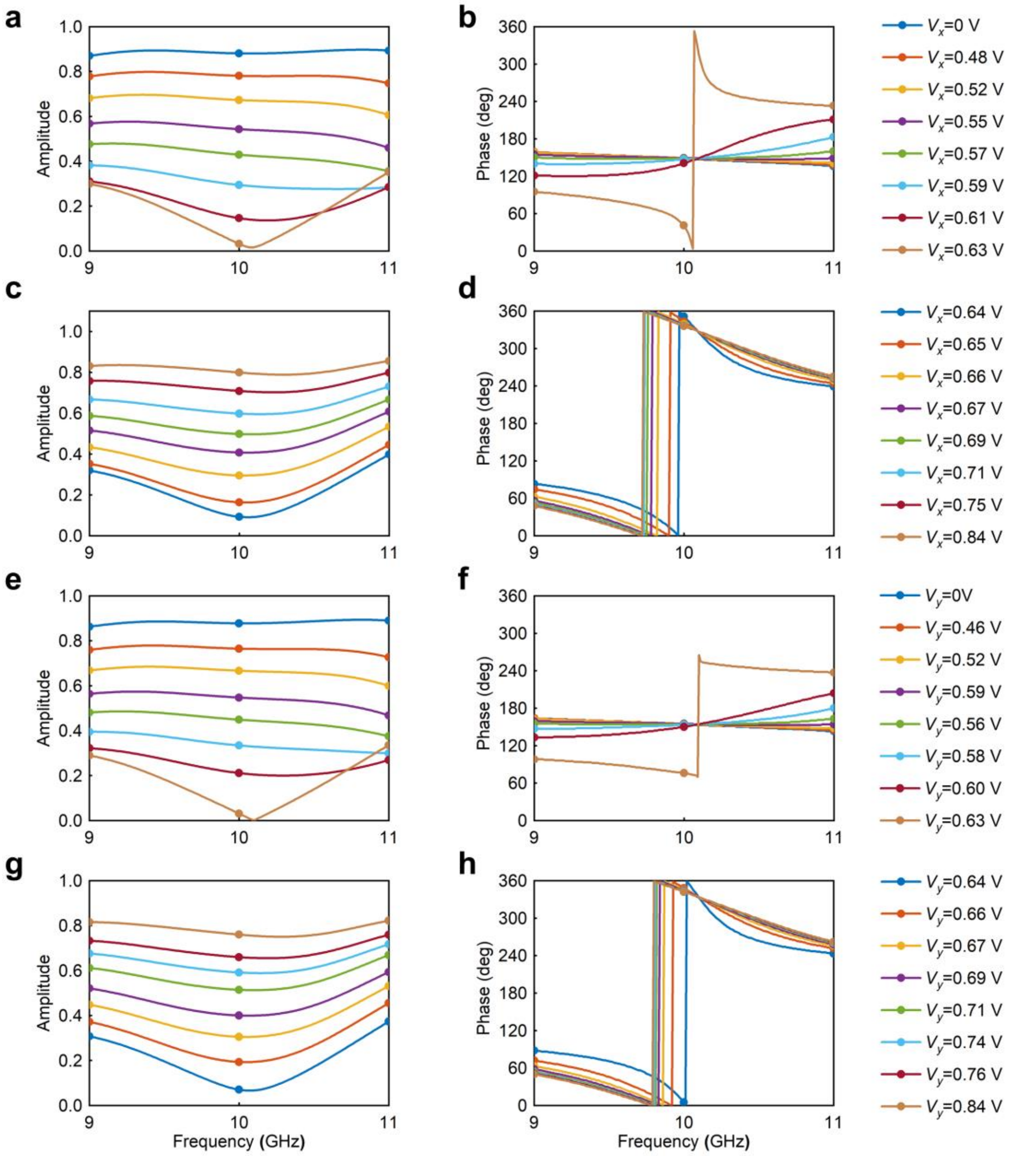

Figure S4. The measured reflection amplitude and phase of unit element varying with V_x and V_y in a wide band of 9-11 GHz. a) Measured reflection amplitude of *x*-polarized wave with V_x=0 to 0.63 V. b) Measured reflection phase of *x*-polarized wave with V_x=0 to 0.63 V. c) Measured reflection

amplitude of x-polarized wave with V_x =0.64 to 0.84 V. d) Measured reflection phase of x-polarized wave with V_x =0.64 to 0.84 V. e) Measured reflection amplitude of y-polarized wave with V_y=0 to 0.63 V. f) Measured reflection phase of y-polarized wave with V_y =0.64 to 0.84 V. g) Measured reflection amplitude of y-polarized wave with V_y=0 to 0.63 V. h) Measured reflection phase of y-polarized wave with V_y =0.64 to 0.84 V.

S5. Measured 2D far-field radiation reflected patterns for four different polarization states

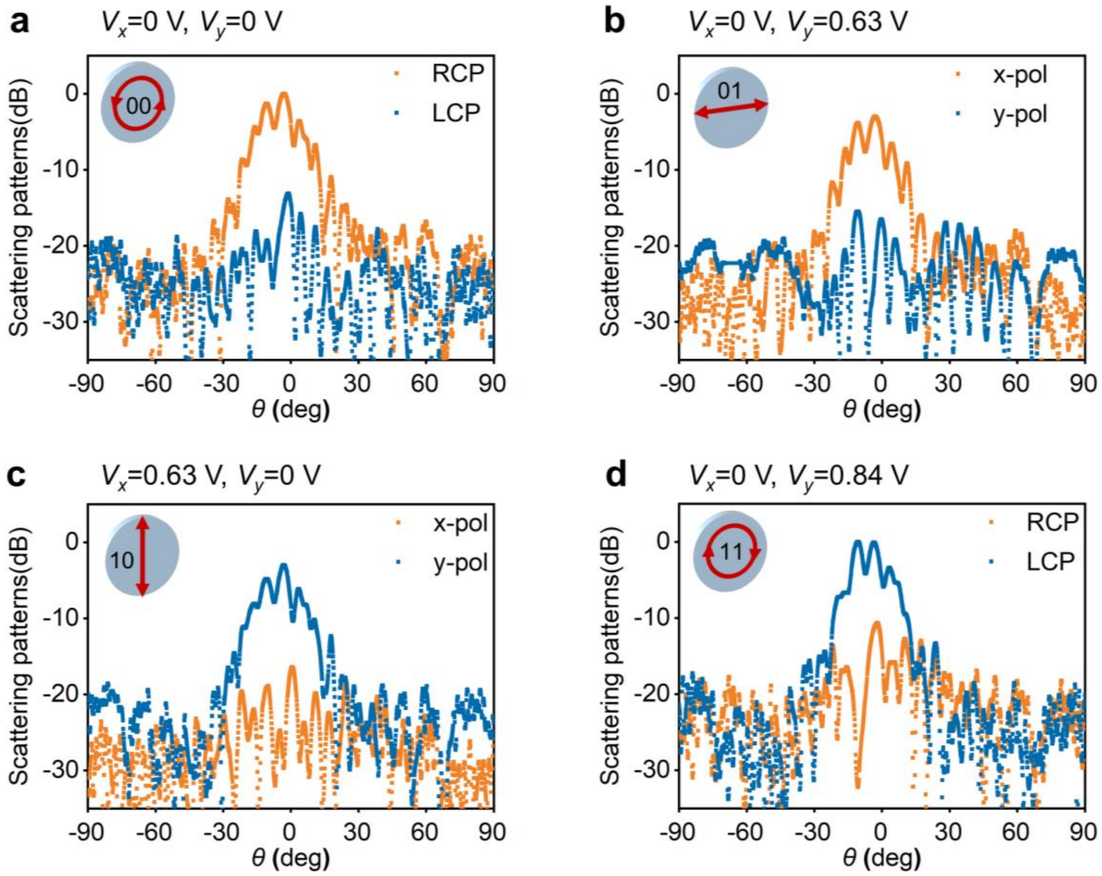

Figure S5. a-d) Measured 2D far-field radiation reflected patterns of a) right-handed circular polarization (RCP), b) x polarization, c) y polarization, and d) left-handed circular polarization (LCP).

The measured far-field radiation patterns of different polarized reflected waves under different bias voltages as shown in Figures S5a-d. When bias voltages of PIN diodes along x and y directions are set to V_x/V_y=0 V/0 V, 0 V/0.63 V, 0.63 V/0 V, and 0 V/0.84 V, the reflected wave will be RCP, x polarization, y polarization, and LCP, respectively.

S6. Polarization discrimination antenna (PDA) and its simulation results

Figures S6a and S6b show the top and bottom views of the proposed array antenna, having a total size of 110×110 mm^2. Twelve square patch elements and the feed circuit are arranged in a mirror-symmetric form, as shown in Figure S6a. The slot lines and slot ring are etched on the back of the antenna, as shown in Figure S6b, where four zero-bias Schottky diodes (SMS7621-040LF) were loaded on the slot ring to realize the double-balanced RF multiplier. A commercial dielectric F4B with a relative permittivity of 2.2, a thickness of 0.5 mm, and a loss tangent of 0.001 is adopted in this design. The dimensions of the parameters are *lp*=110mm, *l*1=30mm, *l*2=20.5mm, *l*3=5.5mm, *w*1=9mm, and *ls*=37.7mm. Figure S6c depicts the measured scattering parameter (S-parameter) S11 of the polarization discrimination antenna, which is below −10 dB in the frequency range of 9.9–10.1 GHz. The simulated radiation pattern of the array antenna at 10GHz is demonstrated in Figure S6, showing that the gain is 13dBi.

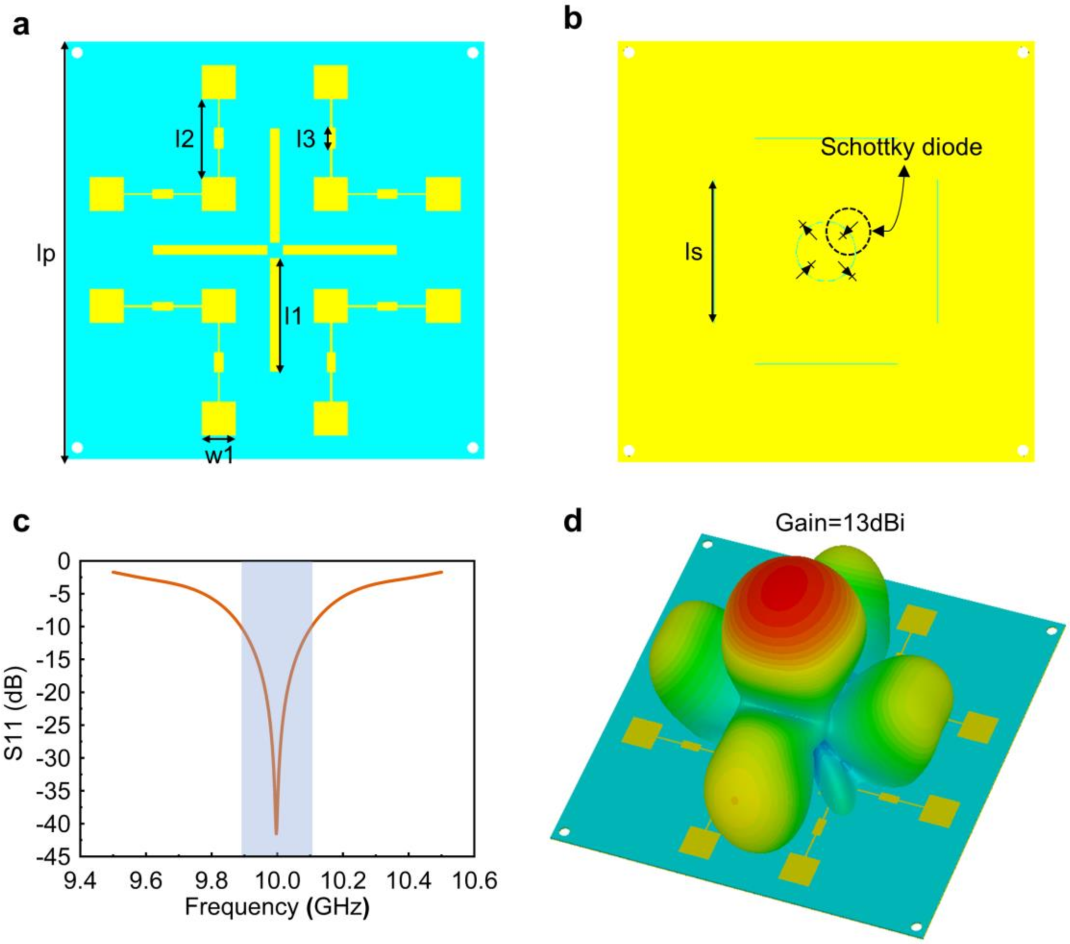

Figure S6. Basic structure of the polarization discrimination patch array antenna for 45°

polarization discrimination. a) Top view of the antenna, b) bottom view of the antenna, and c) S11 of the polarization discrimination antenna. d) Radiation pattern, and gain of the array antenna at 10 GHz.

S7. Fault-tolerant performance of meta-key.

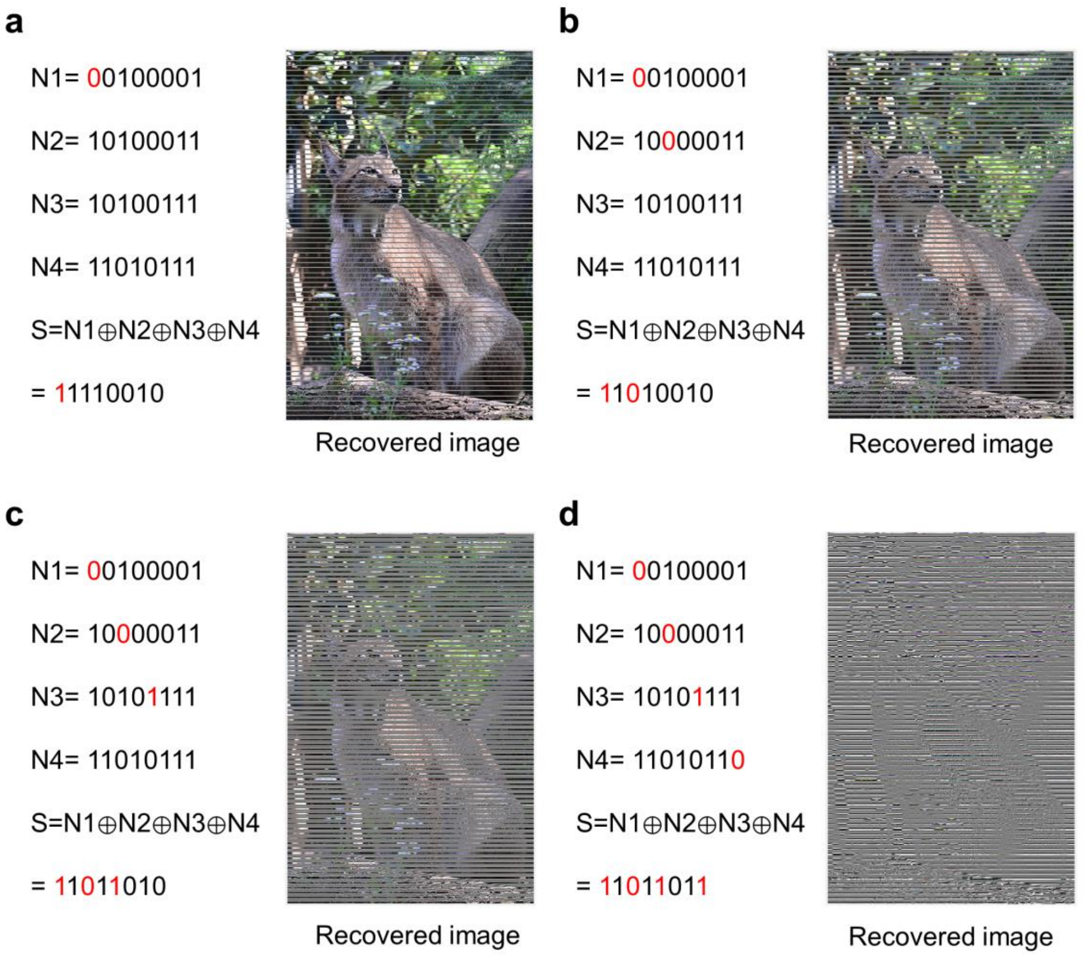

Figure S7. Recovered image with wrong meta-key. a) One error code. b) Two error codes. c) Three error codes. d) Four error codes. Photo credit: Hai Lin Wang, Southeast University.
